# Microglia limit brain tumor development by restricting tumor cell proliferation and inducing T‐cell immunity

**DOI:** 10.1002/1878-0261.70102

**Published:** 2025-08-01

**Authors:** Tzu‐Chieh Sun, Ching‐Fang Yu, Sheng‐Yan Wu, Wei‐Chung Cheng, Chi‐Shiun Chiang, Fang‐Hsin Chen

**Affiliations:** ^1^ Department of Biomedical Engineering and Environmental Sciences National Tsing‐Hua University Hsinchu Taiwan; ^2^ Boron Neutron Capture Therapy Center National Tsing‐Hua University Hsinchu Taiwan; ^3^ Department of Medical Imaging and Radiological Sciences Chang Gung University Taoyuan Taiwan; ^4^ Research Center for Radiation Medicine Taoyuan Taiwan; ^5^ Department of Radiation Oncology Chang Gung Memorial Hospital Linkou Branch Taoyuan Taiwan; ^6^ Ph.D. Program for Cancer Biology and Drug Discovery China Medical University Taichung Taiwan; ^7^ Cancer Biology and Precision Therapeutics Center China Medical University Taichung Taiwan; ^8^ Institute of Nuclear Engineering and Science National Tsing‐Hua University Hsinchu Taiwan; ^9^ Nuclear Science and Technology Development Center National Tsing‐Hua University Hsinchu Taiwan

**Keywords:** glioma, macrophage, microglia, T‐cell immunity

## Abstract

Tumor‐associated macrophages (TAMs) in brain tumors contain two types of macrophages: tumor‐associated microglia and infiltrating macrophages. This study explored whether these two populations have the same role in brain tumor progression. In an *in vitro* coculture model using the astrocytoma cells ALTS1C1 with either the microglial cell line BV2 or the peripheral macrophage cell line RAW264.7, only BV2, not RAW264.7, gathers ALTS1C1 into tumor cell clusters. These BV2‐associated clusters limited ALTS1C1 proliferation but not BV2 cell growth. The *in vivo* studies show that the survival time of mice co‐inoculated with ALTS1C1 and BV2 was prolonged from 30.4 ± 3.1 days to more than 77 days in immune‐competent mice but not in immune‐compromised mice. Examining the tumor microenvironment (TME) by immunohistochemical staining revealed that the co‐inoculation of BV2 increased the CD8 T cells' infiltration and the expression of Granzyme B. Mice bearing with BV2‐containing ALTS1C1 tumor exhibited a reduced level of circulating myeloid‐derived suppressor cells (MDSCs) and an elevated level of CD8 T cells in peripheral blood compared to the ALTS1C1 tumor‐bearing group. This study suggests tumor‐associated microglia restrict brain tumor development by limiting tumor cell proliferation and inducing T‐cell‐associated antitumor immunity.

## Graphical abstract

Microglia (BV2) and IL‐4‐treated BMDMs promote astrocytoma clustering and inhibit tumor growth, not shown in other macrophage cells. *In vivo*, microglial co‐implantation enhances CD8^+^ T cell infiltration, elevates Granzyme B, lowers circulating MDSCs, and extends survival only in immune‐competent mice, but not in immune‐deficient mice. This indicates microglia‐mediated tumor suppression relies on adaptive immunity in the brain tumor microenvironment.

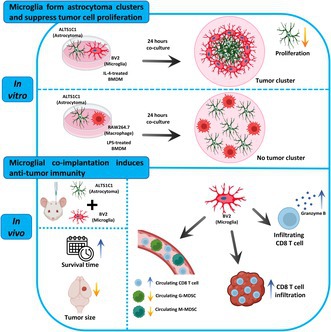

**Abbreviations**
BMDMs: bone‐marrow‐derived macrophages
G‐MDSCs: granulocyte‐like myeloid‐derived suppressor cells
HGG: high‐grade glioma
IF: immunofluorescent
M‐MDSCs: monocyte‐like myeloid‐derived suppressor cells
TAMs: tumor‐associated macrophages
TME: tumor microenvironment

## 1 Introduction

Gliomas, the most common brain tumors, make up about 84.5% of malignant intracranial tumors [1]. According to the WHO classification, there are four levels of glioma, depending on the degree of malignancy [2]. Grade 3 and Grade 4 gliomas are defined as high‐grade gliomas (HGG), carrying a poor prognosis. Despite the advances in treatment options, the median survival time of HGG is merely 18 months [3]. TAMs play a crucial role in glioma progression and treatment response, with their density and location linked to survival outcomes [4, 5, 6].

Tumor‐associated macrophages (TAMs) in brain tumors include resident microglia and infiltrating macrophages [7]. Microglia originate from the yolk sac rather than bone marrow [8, 9] occupy 5–12% of brain cells [10]. Microglia interact with neurons, astrocytes, and oligodendrocytes to maintain homeostasis, address pathology, and regulate tumor development [11]. Microglia have a complex role in tumor progression, showing antitumor functions through phagocytosis and cytotoxic factor release, while also contributing to tumor growth, invasion, and angiogenesis [12, 13, 14]. Additionally, microglia's functions are plastic, as they may secrete toxic factors to suppress tumor cells via Toll‐like receptor 3 induction [15]. Understanding their dual roles in brain tumors can aid in therapeutic strategies for aggressive brain cancer.

Based on their pro‐inflammatory and anti‐inflammatory functions, TAMs are broadly categorized into two subsets: M1 and M2 macrophages. M1 macrophages are activated by lipopolysaccharide (LPS), IFN‐γ, and TNF‐α, while M2 macrophages are polarized by IL‐4, IL‐10, and IL‐13 [16, 17, 18]. M1‐like TAMs exhibit antitumor effects, activating T helper and cytotoxic T cells through cytokines like IL‐12. In contrast, M2‐like TAMs promote tumor growth by inhibiting cytotoxic T‐cell function and interacting with regulatory T cells via IL‐10 [17, 19, 20, 21]. Although the crosstalk between macrophages and adaptive immunity is well documented, it is often extrapolated to microglia, with limited direct evidence [22, 23]. The crosstalk between tumor‐associated microglia and CD8^+^ T cells is a key factor in brain tumor immune response, with microglia inhibiting the cytotoxic function of CD8^+^ T cells [24].

This study explores the distinct roles of resident microglia versus infiltrating macrophages in malignant glioma progression. An *in vitro* coculture model was established to simulate the TME of brain tumor invasion, and an *in vivo* co‐implantation model was employed to examine the effects of microglia on tumor growth. Malignant murine astrocytoma cell lines, ALTS1C1 and ALTS1C1‐GFP, serve as glioma models [25]. The BV2 microglia cell line, representing resident microglia, was chosen for its interactions with T cells and cytokine responses [26]. The RAW264.7 macrophage cells were employed to represent infiltrating macrophages [27, 28]. This study demonstrates that microglial cells, but not infiltrating macrophages, promote astrocytoma cluster formation and limit tumor cell proliferation *in vitro*. *In vivo*, the findings emphasize the role of CD8+ T cells in the microglia‐associated delays in brain tumor growth.

## 2 Materials and methods

### 2.1 Cell culture

The murine astrocytoma cell line ALTS1C1 (RRID: CVCL_Y088) and its GFP‐expressing variant, ALTS1C1‐GFP, created by infecting ALTS1C1 with a lentiviral GFP‐expressing vector, were previously established in our lab [25, 29]. The BV2 microglia cell line (RRID: CVCL_0182) represented resident microglia [26], while RAW264.7 (RRID: CVCL_0493) served as a model for infiltrating macrophages [27, 28]. Both BV2 and RAW264.7 cell lines were purchased from Bioresource Collection and Research Center (BCRC, Hsinchu, Taiwan). To ensure cell line authenticity, all cell lines used in this study were authenticated within the past 3 years by Short Tandem Repeat (STR) profiling, performed by a certified third‐party provider (TopGen Biotechnology, Kaohsiung, Taiwan). STR profiles of BV2 and RAW264.7 cells were cross‐checked against reference profiles in the ExPASy database. For ALTS1C1 cells, identity was confirmed based on genomic DNA sequence data obtained during the initial establishment of the cell line. In addition, ALTS1C1‐GFP cells were routinely verified for GFP expression using fluorescence microscopy prior to experimental use. In addition, mycoplasma detection was performed once a week during cell culture with EZ‐PCR Mycoplasma Detection Kit (Sartorius, Göttingen, Germany) to ensure that the practicing cells are not contaminated by mycoplasma. The cells were cultured with Dulbecco's Modified Eagle Medium (DMEM; Gibco®, Waltham, MA, USA). The fetal bovine serum (FBS; Gibco®) and penicillin–streptomycin (PS; Gibco®) occupied 10% and 1% in the DMEM medium. The cells were maintained at 37 °C and 5% in two humidified air atmospheres. The coculture system was established with a seeding density of 3.2 × 10^3^ cells per mm^2^ on a 4‐well chamber slide (Thermo Fisher Scientific, Waltham, MA, USA) and incubated for 24 h as in our previous study [29].

### 2.2 Bone‐marrow‐derived macrophages (BMDMs) collection, culture, and polarization

Bone marrow was collected from the femurs and tibias of adult mice, flushed with cold Roswell Park Memorial Institute (RPMI; Gibco®) buffer, and collected. The marrow was lysed with RBC buffer (Invitrogen, Carlsbad, CA, USA) on ice for 5 min, then placed in 6‐well dishes (Corning, NY, USA) with RPMI (Gibco®), 10% FBS (Gibco®), and 1% PS (Gibco®). The following day, the supernatant was collected and incubated with granulocyte‐macrophage colony‐stimulating factor (GM‐CSF; R&D System, Minneapolis, MN, USA) before being seeded in low‐attachment dishes (Corning). The medium was refreshed every 2 days until day 7, when BMDMs became confluent. BMDMs were then polarized with 20 ng·mL^−1^ IL‐4 (R&D Systems) or 200 ng·mL^−1^ LPS (LPS; Sigma, Burlington, MA, USA).

### 2.3 Cell RNA sequencing and analysis

RNA was extracted from BV2 and RAW264.7 cells using TRIzol reagent, then mixed with 1‐bromo‐3‐chloropropane (BCP; Sigma‐Aldrich) (TRIzol: BCP = 5 : 1) and incubated at room temperature for several minutes. After centrifugation at 12 000 ***g***, the supernatant was mixed with isopropanol for RNA precipitation. Following another centrifugation at 12 000 ***g***, the supernatant was removed, and RNA was washed with 1 mL of 75% DEPC‐treated ethanol at 7500 ***g*** for 5 min. The RNA pellet was air‐dried, suspended in DEPC‐treated water, and stored at −80 °C. Phalanx Biotech performed RNA quality and bulk sequencing. M1 and M2‐associated genes were analyzed by incorporating sequencing data from a reference article with M1 and M2‐related genes [30].

### 2.4 Cell cycle test

3 × 10^5^ ALTS1C1‐GFP and the corresponding ratio of BV2 were seeded on 12‐well plates (Corning), and cells were cultured for 24 h. Afterward, cells were fixed in methanol (Honeywell, Charlotte, NC, USA) at $-20^{{}^{\circ}}C$ for 10 min, then treated with 0.1% Triton X‐100 (Sigma) for 10 min. Cells were stained with a PI solution containing 40 μg·mL^−1^ Propidium Iodide (Sigma), and 200 μg·mL^−1^ RNase (Thermo Fisher Scientific) for 10 min at room temperature before flow cytometry analysis. The cell cycle data were analyzed using the Dean‐Jett‐Fox model.

### 2.5 Animal procedures

6 to 8‐week‐old C57BL/6J and C.B17 male mice were purchased from the National Laboratory Animal Center of Taiwan. This study was approved by the Institutional Animal Care and Use Committee (IACUC) of National Tsing Hua University, Taiwan (IACUC approval No. 111029). Mice were handled under, 21~23 °C and a humidity of 50% ~ 60%. Mice were housed in an Individually Ventilated Cage System (IVC System) under a 12‐h light 12‐dark cycle, and the light intensity was lower than 300 Lux. Mice were anesthetized with a mixture of 25% IMALGENE 1000 (Merial Laboratoire de Toulouse, Toulouse, France) and 25% Rompun®2% (Bayer HealthCare Animal Health, Shawnee, KS, USA) via intraperitoneal (I.P.) injection for surgeries. When the tumor‐bearing mice arched their back, and the body weight was lower than 17 grams, 25% Urethane (Sigma) in PBS was used for euthanasia.

### 2.6 Orthotopic brain tumor implantation

$5\times 10^{4}$ ALTS1C1 or ALTS1C1‐GFP cells were mixed with the corresponding ratio of BV2 cells in a 2μL volume of DMEM medium. The implanted location was 1.0 mm posterior to bregma and 2.0 mm lateral to the midline of the skull. The skull was drilled into a tiny hole for tumor cell injection to a depth of 2.5 mm. Then, the holes on the skull were filled with bone wax (ETHICON, Raritan, NJ, USA), and the mice's skin was stitched.

### 2.7 Intramuscular tumor implantation and tumor size measuring

The cells suspended in 100 μL PBS were injected into the muscle of the left hind leg of the mice. The tumor sizes were measured with a caliper every day until the experiment was completed. Furthermore, the tumor sizes were determined by calculating the average of the horizontal and vertical diameters.

### 2.8 Tissue collection and frozen section

At 10 and 24 days post‐tumor implantation, mice were euthanized, and their cardiovascular systems were perfused with a solution of 4% paraformaldehyde (Sigma) dissolved in PBS. The brain tissue was carefully buried in the OCT compound (Optimal Cutting Temperature; Sakura, Torrance, CA, USA) and stored in the $-80^{{}^{\circ}}C$ refrigerator. Frozen tissue was sectioned into10μm with a cryostat (Leica, Wetzlar, Germany) and adhered to the salinized slide (DAKO, Glostrup, Denmark).

### 2.9 H&E staining

The tissue slide was fixed with methanol (Honeywell) at −20 °C for 10 min. The fixed tissue was soaked in Hematoxylin solution (Merck, Darmstadt, Germany) for 1 min and then immersed in 0.25% ammonium hydroxide (Merck) for 10 s. Second, the tissue slide was embedded into Eosin Y‐solution (Merck), containing 0.2% acetic acid for 20 s. The images were taken with an inverted microscope (ZEISS, Oberkochen, Germany), and the tumor size was quantified by Image‐Pro Plus 6.0 software (Media Cybernetics, Rockville, MA, USA).

### 2.10 Cells and brain tissue immunofluorescence (IF) staining

Cells or frozen tissue were fixed with methanol at −20 °C for 10 min and soaked in 0.1% Triton (Sigma) for 10 min. Then, the fixed specimens were blocked with 4% FBS and 1% goat serum at room temperature for 1 h. The specimens were stained with the primary antibodies of purified rat anti‐mouse CD11b (BD Biosciences, Franklin Lakes, NJ, USA), purified rat anti‐mouse CD8a (BD Biosciences), purified rat anti‐mouse anti‐Granzyme B (Abcam, Cambridge, UK), and CD4 (BD Biosciences) overnight at 4 °C. The next day, the secondary antibody Alexa Fluor 594 donkey anti‐rabbit IgG(H + L) (Thermo Fisher Scientific) and Alexa Fluor 647 donkey anti‐rat IgG(H + L) (Thermo Fisher Scientific) were applied at room temperature for 1 h. Finally, the tissue was stained with DAPI. The quantification of signal expression was performed using Image‐Pro Plus 6.0 software.

### 2.11 Circulating T cells and MDSCs collection and analysis

The peripheral blood was extracted and mixed with 1XRBC Lysis Buffer (Invitrogen eBioscience, Carlsbad, CA, USA) to lyse the red blood cells at room temperature for 10 min. Subsequently, the RBC‐lysed blood samples were blocked with a blocking buffer (Fc block (BD Biosciences): goat serum: PBS = 1 : 5 : 500) on ice for 30 min. Finally, the blocked samples were stained with antibodies of PE‐Cy7 rat anti‐mouse CD45 (BD Biosciences), APC rat anti‐mouse CD8a (BD Biosciences), FITC rat anti‐mouse Ly6C (BD Biosciences), and PE rat anti‐mouse Ly6G (BD Biosciences). FACS analysis was performed on the Canto cytometer (BD Bioscience), and data were analyzed by FlowJo software.

### 2.12 Time‐lapse video recordation

The process of cell co‐culturing was meticulously monitored through time‐lapse imaging using the automated inverted microscope (Ti‐Eclipse; Nikon, Tokyo, Japan) and the ORCA‐Flash4.0 camera (Hamamatsu, Shizuoka Pref, Japan), both housed within an onstage incubator to maintain consistent culture conditions.

### 2.13 Statistics

Quantitative analysis was conducted using Prism software 8.0 (GraphPad). Significance was assessed using the *t*‐test for quantitative data and the Log‐rank test for survival curves. Results were considered significant when the *P*‐value was < 0.05.

## 3 Result

### 3.1 Microglial cell line renders ALTS1C1 to form the clusters and limits the proliferation of ALTS1C1 *in vitro*

**Fig. 1:**
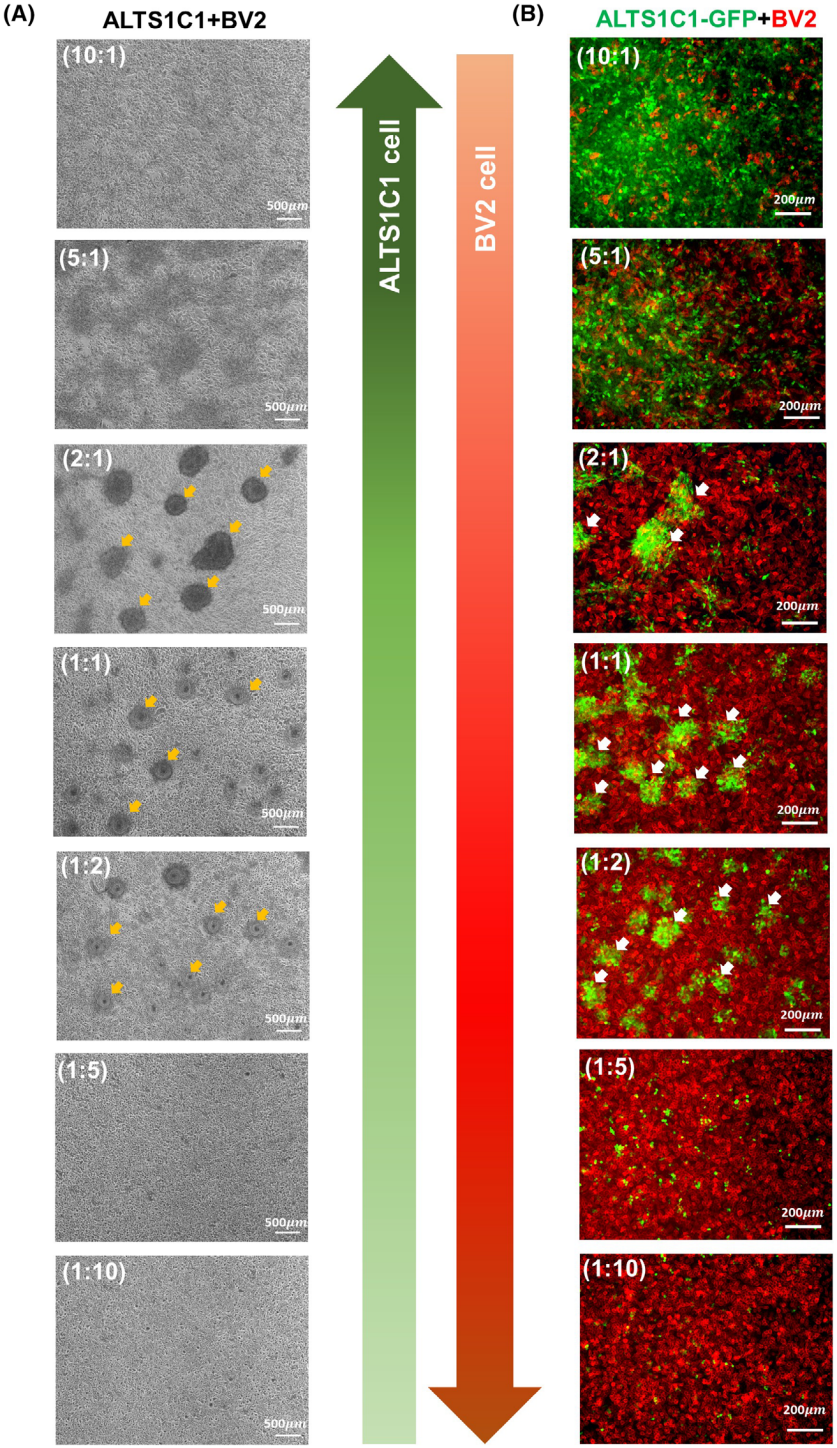
BV2 cell density determines ALTS1C1 tumor cluster formation and spatial organization in coculture (A) Representative images of cocultures of ALTS1C1 astrocytoma cells and BV2 microglia at varying ratios (ALTS1C1:BV2 = 10 : 1, 5 : 1, 2 : 1, 1 : 1, 1 : 2, 1 : 5, and 1 : 10) after 24 h of incubation. The total cell density was maintained at 3.2 × 10^3^ cells per mm^2^. Tumor clusters (diameter > 50 μm) were observed specifically at ratios of 2 : 1, 1 : 1, and 1 : 2, as indicated by yellow arrows. Scale bar = 500 μm. Each condition was independently repeated three times (*n* = 3). (B) Representative images of cocultures of ALTS1C1‐GFP (green) and BV2 (stained with myeloid cell marker CD11b, red) at varying ratios (ALTS1C1:BV2 = 10 : 1, 5 : 1, 2 : 1, 1 : 1, 1 : 2, 1 : 5, and 1 : 10) after 24 h of incubation. White arrows indicated the clusters. Scale bar = 200 μm. Each condition was independently repeated three times (*n* = 3).

**Fig. 2:**
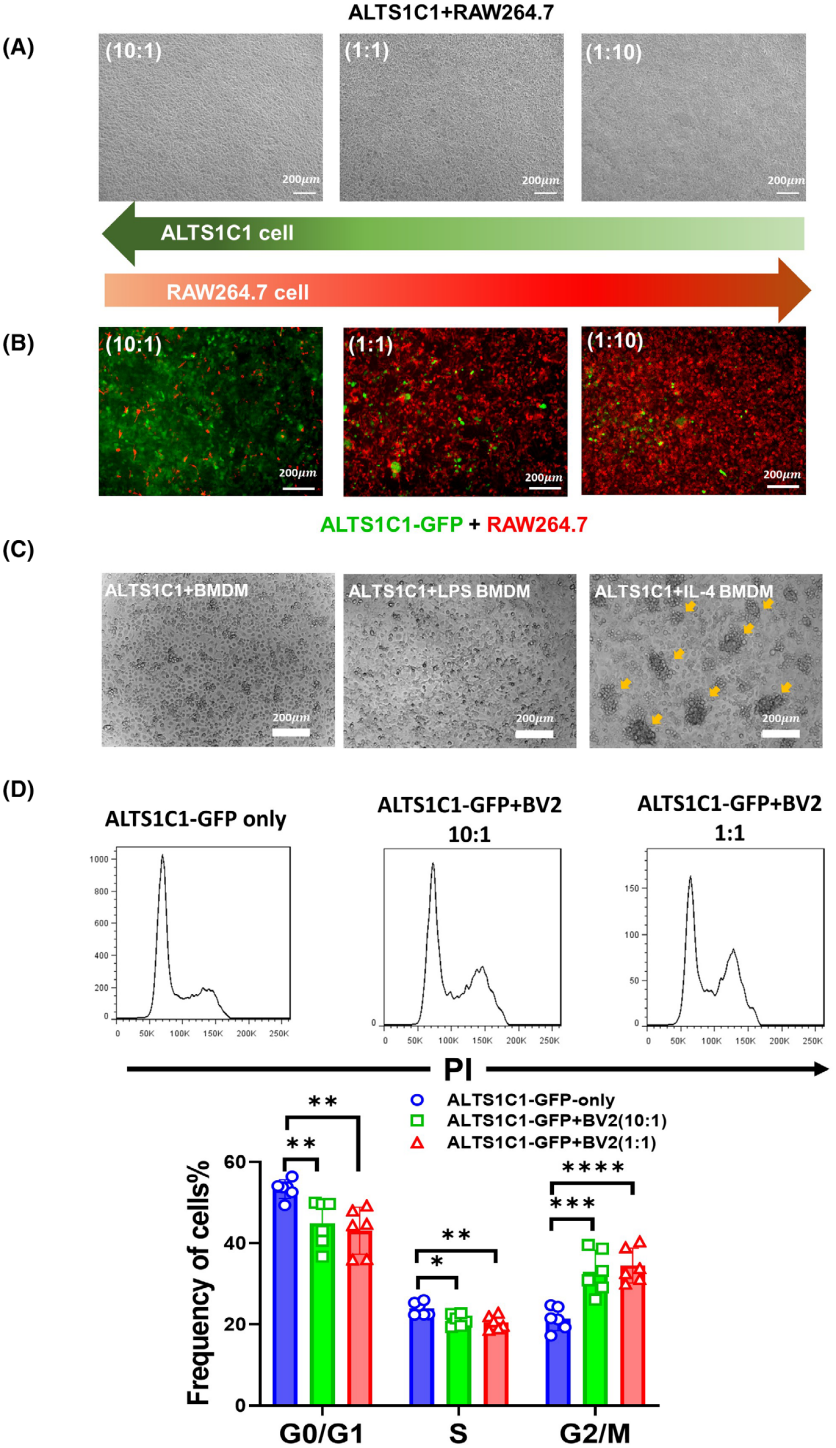
BV2 but not RAW264.7 or LPS‐Treated BMDMs induce ALTS1C1 cluster formation and G2/M cell cycle arrest (A) Representative images of cocultures of ALTS1C1 astrocytoma cells and RAW264.7 macrophage at varying ratios (ALTS1C1:RAW264.7 = 10 : 1, 1 : 1, and 1 : 10) after 24 h of incubation. The total cell density was maintained at 3.2 × 10^3^ cells per mm^2^. Scale bar = 200 μm. Each condition was independently repeated three times (*n* = 3). (B) Representative images of cocultures of ALTS1C1‐GFP (green) and RAW264.7 (stained with myeloid cell marker CD11b, red) at varying ratios (ALTS1C1:BV2 = 10 : 1, 1 : 1, and 1 : 10) after 24 h of incubation. Scale bar = 200 μm. Each condition was independently repeated three times (*n* = 3). (C) Representative images of cocultures of ALTS1C1 astrocytoma cells with BMDMs, LPS‐treated BMDMs, or IL‐4‐treated BMDMs at a 1 : 1 ratio after 24 h of incubation. The total cell density was maintained at 3.2 × 10^3^ cells per mm^2^. Tumor clusters (diameter > 50 μm) are specifically observed in coculture of ALTS1C1 and IL‐4‐treated BMDMs, as indicated by yellow arrows. Scale bar = 200 μm. Each condition was independently repeated three times (*n* = 3). (D) Cell cycle analysis of ALTS1C1‐GFP cells after 24 h of culture alone or coculture with BV2 microglia at different ratios (ALTS1C1‐GFP:BV2 = 10 : 1 and 1 : 1), using PI staining and flow cytometry. Data are presented as mean ± SD (*n* = 6). Statistical analysis was performed using unpaired *t*‐tests (*0.01 < *P* < 0.05, **0.001 < *P* < 0.01, ***0.0001 < *P* < 0.001, ****P < 0.0001). A significantly higher proportion of ALTS1C1‐GFP cells were arrested in the G2/M phase during coculture with BV2.

Previous studies have reported that microglia are the primary components of the TME in invasive brain tumors [25]. To investigate the role of microglia in tumor invasion, an *in vitro* coculture model was established using the BV2 microglial cell line and the ALTS1C1 astrocytoma cell line. Our previous study found that BV2 cells rendered ALTS1C1 into tumor cell clusters and increased the chemoresistance of ALTS1C1 cells when cocultured in a 1 : 1 ratio [29]. This study further investigated the cluster formation phenomena by co‐culturing BV2 and ALTS1C1 cells at different ratios (Fig. 1A). The results revealed that a critical density of both ALTS1C1 cells and BV2 is required for ALTS1C1 cells to form clusters. When the density of ALTS1C1 was decreased to one‐sixth of the total cell population (1 : 5 and 1 : 10 ratios) or the density of BV2 was reduced to one‐fifth of ALTS1C1 cells (5 : 1 and 10 : 1 ratios), the ALTS1C1 clusters failed to form. To further investigate the spatial distribution of these two cells, ALTS1C1‐GFP cells were used to replace ALTS1C1. The ALTS1C1‐GFP cells exhibited the same growth rate as ALTS1C1 cells during culture (Fig. S1A). Immunofluorescent (IF) imaging using the CD11b marker to stain BV2 cells revealed a distinct spatial distribution, with ALTS1C1‐GFP cells primarily occupying the core of the cluster and BV2 cells surrounding the periphery (Fig. 1B, Fig. S1B). The time‐lapse videos showed that ALTS1C1 cells were forced to move together in the presence of BV2 cells (Fig. S1C). To investigate whether this is a unique feature of microglia or a common feature of macrophages, a common macrophage cell line, such as RAW264.7, was used in the same coculture system. No clusters formed at any ratio of RAW264.7 and ALTS1C1 coculture (Fig. 2A). The IF image and time‐lapse videos further demonstrate a homogeneous distribution between RAW264.7 and ALTS1C1‐GFP during 24 h of coculture (Fig. 2B, Fig. S1C). Gene profiling revealed that BV2 cells expressed higher levels of both M2‐associated genes (Maf, Selenop, P2ry14, Mrc1, Ctsc, and Cxcr4) and M1‐associated genes (Il7r, Atf3, and Gadd45g) compared to RAW264.7 cells (Fig. S2A), suggesting its multifaceted role in interaction with brain tumors. To confirm the relationship between ALTS1C1 cluster formation and the function of macrophages, the coculture system was further examined with polarized BMDMs (Fig. S2B). Clusters formed when macrophages were replaced by IL‐4, not LPS‐treated BMDMs (Fig. 2C). We hypothesize that BV2 may have a similar function to IL‐4‐treated BMDMs to limit ALTS1C1 tumor cell proliferation. Coculture analysis showed BV2 cells altered ALTS1C1‐GFP cell cycles, reducing the G0/G1 phase from 53% to 45% and the S phase from 24% to 20%, while increasing the G2/M phase from 23% to 35%, suggesting G2/M arrest and growth inhibition of tumor cells in BV2‐ALTS1C1‐GFP clusters (Fig. 2D).

### 3.2 BV2 restricts ALTS1C1 tumor growth *in vivo*

**Fig. 3:**
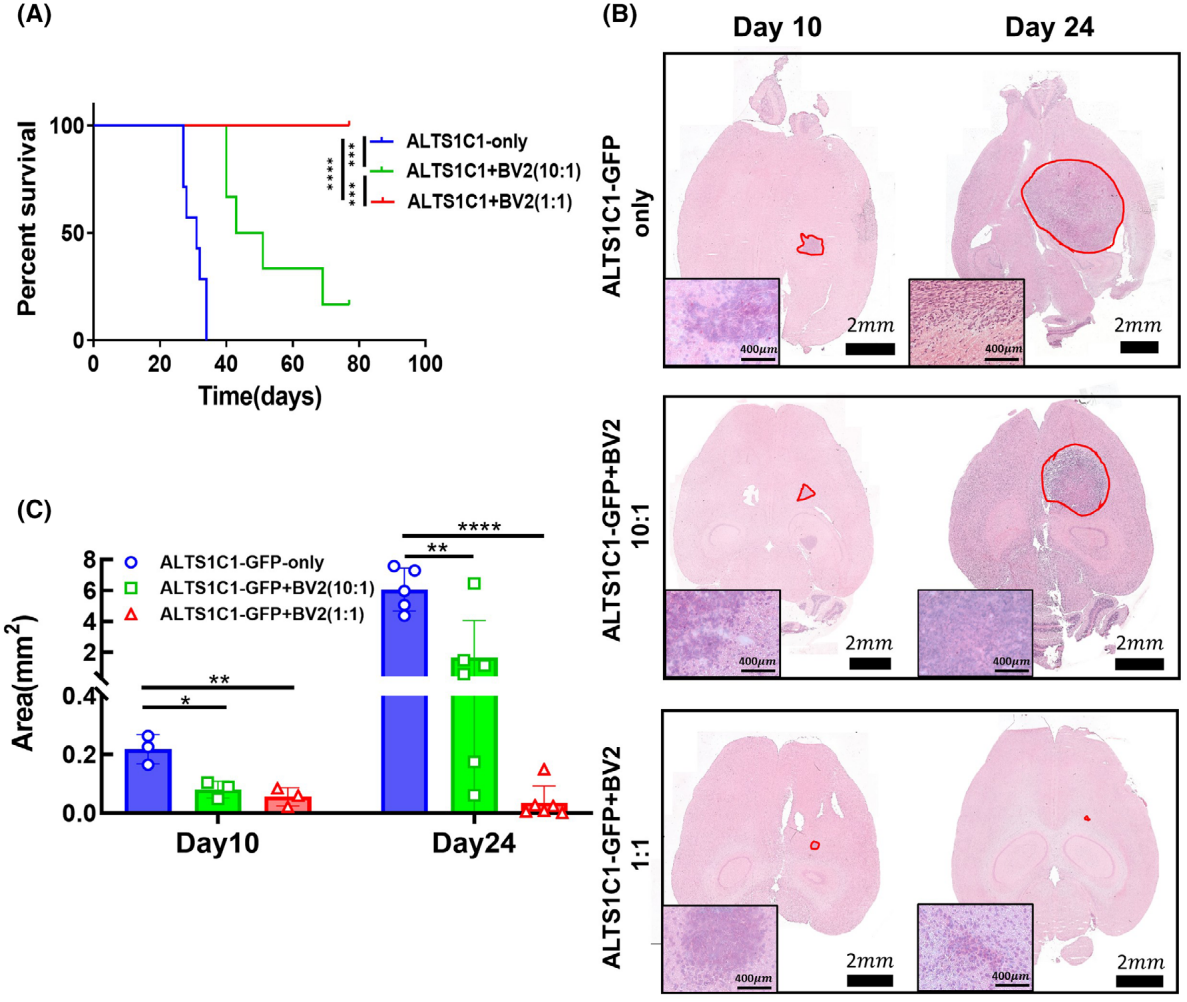
BV2 restricts ALTS1C1 tumor growth *in vivo* (A) Kaplan–Meier survival curves of C57BL/6 immunocompetent mice bearing ALTS1C1 tumors. Groups included ALTS1C1 only (*n* = 7), ALTS1C1 + BV2 (10 : 1) (*n* = 6), and ALTS1C1 + BV2 (1 : 1) (*n* = 10). Survival was analyzed using the log‐rank (Mantel–Cox) test (***0.0001 < *P* < 0.001, ****P < 0.0001). Co‐injection with BV2 significantly prolonged the survival time in a cell number‐dependent manner. (B) Representative H&E‐stained images of ALTS1C1‐GFP astrocytoma within brain tissue of C57BL/6 mice from three groups: ALTS1C1‐GFP only, ALTS1C1‐GFP + BV2 (10 : 1), and ALTS1C1‐GFP + BV2 (1 : 1). Brain tissues were collected on day 10 and day 24 after tumor implantation. Tumor regions are outlined in red. Scale bars: 2 mm (whole brain), 400 μm (magnified tumor region in inset). (C) Quantification of the largest tumor section area of ALTS1C1‐GFP astrocytoma in brain tissue of C57BL/6 mice from three groups—ALTS1C1‐GFP only, ALTS1C1‐GFP + BV2 (10 : 1), and ALTS1C1‐GFP + BV2 (1 : 1)—on days 10 and 24 after tumor implantation. Data are presented as mean ± SD (*n* ≥ 3). Statistical analysis was performed using unpaired *t*‐tests (*0.01 < *P* < 0.05, **0.001 < *P* < 0.01, ****P < 0.0001). Tumors co‐injected with BV2 cells were significantly smaller than those in the ALTS1C1‐GFP only group.

*In vitro* experiments demonstrated that BV2 cells could render ALTS1C1 tumor cells to form clusters, limiting tumor cell proliferation. To assess whether BV2 could also limit astrocytoma growth *in vivo*, immunocompetent C57BL/6 mice were orthotopically co‐inoculated with ALTS1C1 and BV2 cells. In the ALTS1C1‐only group (5 × 10^4^ ALTS1C1 cells), the mean survival time of tumor‐bearing mice was 30.4 ± 3.1 days. On the other hand, BV2 cells (up to 1 × 10^6^ cells) could not form tumors in the brain, and thus no tumor growth data could be presented. All mice co‐inoculated with equal numbers of ALTS1C1 and BV2 cells (5 × 10^4^ of each) survived up to at least 77 days (Fig. 3A), with no detectable tumors at this time. When the number of BV2 cells was reduced to one‐tenth of the ALTS1C1 cells (5 × 10^4^ ALTS1C1 cells +5 × 10^3^ BV2 cells), the mean survival of tumor‐bearing mice decreased to 48.6 ± 12.3 days, which remained significantly longer than the ALTS1C1‐only group. These results underscore the significant role of BV2 in limiting ALTS1C1 tumor cell growth *in vivo*. A similar conclusion was obtained in the intramuscular tumor model (Fig. S3A). To further examine the impact of BV2 on astrocytoma progression, the tumor size within ALTS1C1‐GFP‐bearing mice was analyzed at 10‐ and 24‐day post‐tumor implantation. To improve the identification of small tumors during tissue slicing, we used ALTS1C1‐GFP instead of ALTS1C1 in the following tumor size quantification experiment. The GFP fluorescence signal allowed for the precise localization of microtumor lesions under a microscope. Importantly, despite ALTS1C1 cells being transfected with GFP, previous tumor growth assays showed that the GFP transfection did not affect tumor growth *in vivo* (Fig. S3B,C). H&E staining shows that tumor size decreased with the increasing BV2 ratio (Fig. 3B). Quantification data (Fig. 3C) revealed that the presence of BV2 resulted in significantly smaller ALTS1C1‐GFP tumor sizes than the ALTS1C1‐GFP‐only group as a function of the BV2 ratio. These findings suggest that BV2 restrains the progression of astrocytoma in a BV2 number‐dependent manner. To further examine the change in tumor size, results (Fig. 3C) indicated that tumors continue to grow from day 10 to 24 in both ALTS1C1‐GFP‐only and ALTS1C1‐GFP containing one‐tenth BV2 groups, but the tumor size of ALTS1C1‐GFP containing the same number of BV2 group at 24 days did not get bigger than that at 10 days, even shrinking. This result indicates factors other than cell proliferation inhibition are involved in BV2‐mediated tumor growth delay.

### 3.3 BV2‐mediated ALTS1C1 tumor growth delay depends on host immunity

**Fig. 4:**
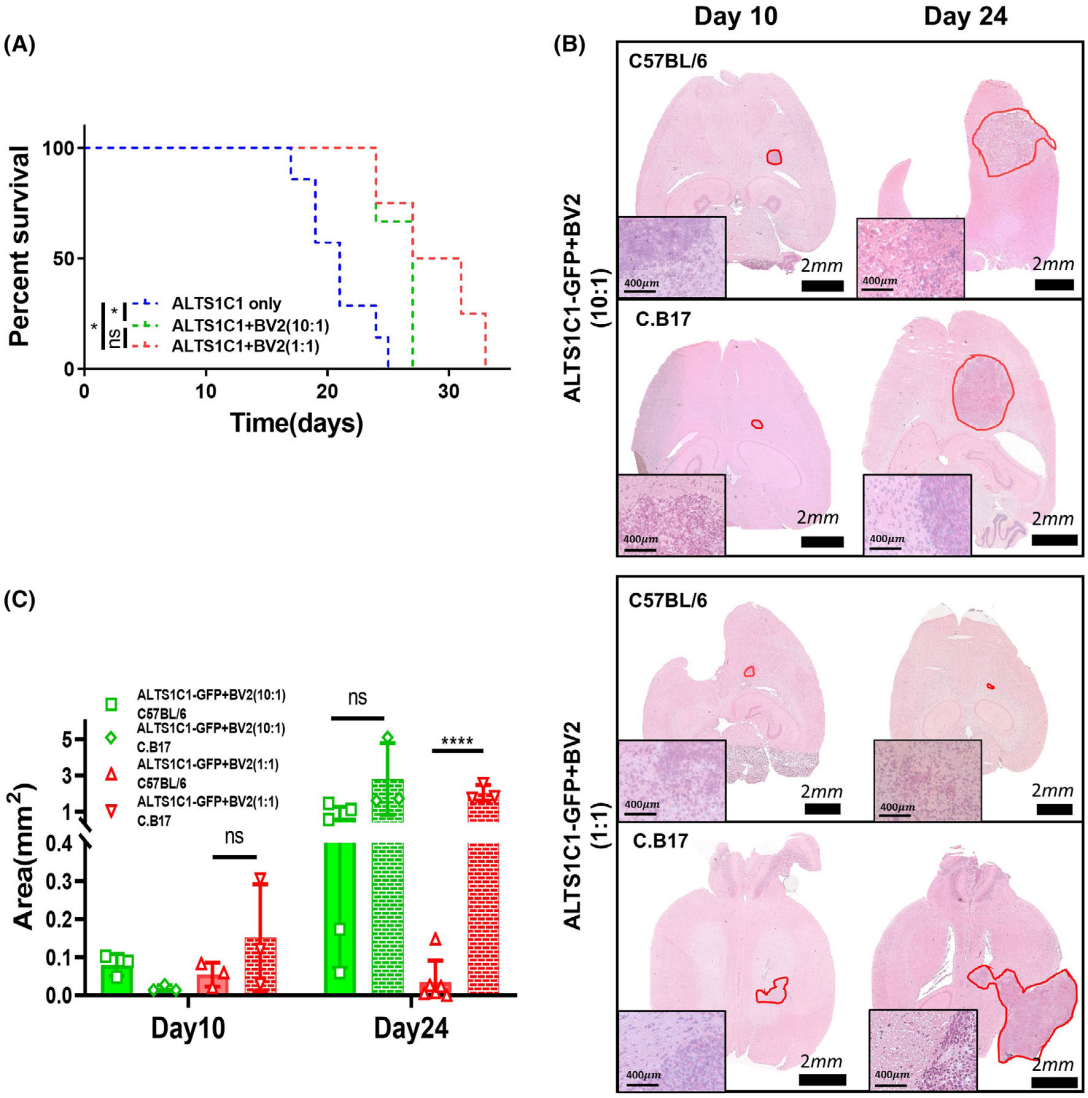
BV2‐mediated ALTS1C1 tumor growth delay depends on host immunity (A) Kaplan–Meier survival curves of C.B17 SCID mice bearing ALTS1C1 tumors. Groups included ALTS1C1 only (*n* = 7), ALTS1C1 + BV2 (10 : 1) (*n* = 3), and ALTS1C1 + BV2 (1 : 1) (*n* = 4). Survival was analyzed using the log‐rank (Mantel–Cox) test (ns, not significant, *0.01 < *P* < 0.05). Co‐injection with BV2 cells significantly prolonged survival; however, the cell number‐dependent effect observed in immunocompetent mice was not maintained in SCID mice. (B) Representative H&E‐stained images of ALTS1C1‐GFP astrocytoma in brain tissue of C57BL/6 and C.B17 mice co‐injected with BV2 cells at ratios of 10 : 1 or 1 : 1. Brain tissues were collected on days 10 and 24 after tumor implantation. Tumor regions are outlined in red. Scale bars: 2 mm (whole brain), 400 μm (magnified tumor region in inset). (C) Quantification of the largest tumor section area of ALTS1C1‐GFP astrocytoma in brain tissue of C57BL/6 and C.B17 mice co‐injected with BV2 cells at ratios of 10 : 1 or 1 : 1. Brain tissues were collected on days 10 and 24 after tumor implantation. Data are presented as mean ± SD (*n* ≥ 3). Statistical analysis was performed using unpaired *t*‐tests (ns, not significant, ****P < 0.0001). The tumor growth restriction observed in C57BL/6 mice co‐injected with ALTS1C1‐GFP and BV2 (1 : 1) was not observed in C.B17 mice, suggesting a role of the immune system in mediating BV2‐induced tumor suppression.

The tumor size decreased after 10 days in the group containing more BV2 cells, indicating that the cell proliferation rate may not be the only factor for BV2‐mediated tumor growth delay. The hypothesis was that host immunity could be involved. To investigate this further, a T‐cell‐deficient C.B17 SCID mouse model was used. Remarkably, SCID mice solely implanted with ALTS1C1 tumor cells had significantly shorter mean survival (21.1 ± 3.0 days) (Fig. 4A) compared to C57BL/6 mice (30.4 ± 3.1 days) (Fig. 3A), highlighting the crucial role of T cells in tumor progression. In SCID mice, the BV2‐mediated delay in tumor growth was diminished (Fig. 4A). The Kaplan–Meier survival curves show that C.B17 SCID mice inoculated with the same number of ALTS1C1 and BV2 cells (1 : 1 ratio) had a mean surviving day of 28.8 ± 4.0 days (Fig. 4A), even shorter than the mean surviving day of ALTS1C1‐only grown in C57BL/6J mice. When the number of BV2 cells was further decreased to one‐tenth of ALTS1C1 cells, survival times in SCID mice (26.0 ± 1.7 days) did not differ significantly from that of the 1 : 1 ratio group. To deepen our understanding of tumor progression, mice inoculated with various ratios of ALTS1C1/BV2 cells were sacrificed on day 10 and day 24, and tumor section areas were assessed via H&E staining (Fig. 4B). The quantification of the largest tumor section area (Fig. 4C) reveals that the absence of T cells did not significantly impact tumor size on day 10. However, by day 24, the tumor size in the group of C.B17 mice inoculated with ATLS1C1 and BV2 had become significantly larger than that observed in C57BL/6 mice. This observation signifies the important roles of T cells in BV2‐associated ALTS1C1 growth restriction, and this influence becomes pronounced after 10 days.

### 3.4 The presence of BV2 enhances T‐cell infiltration

**Fig. 5:**
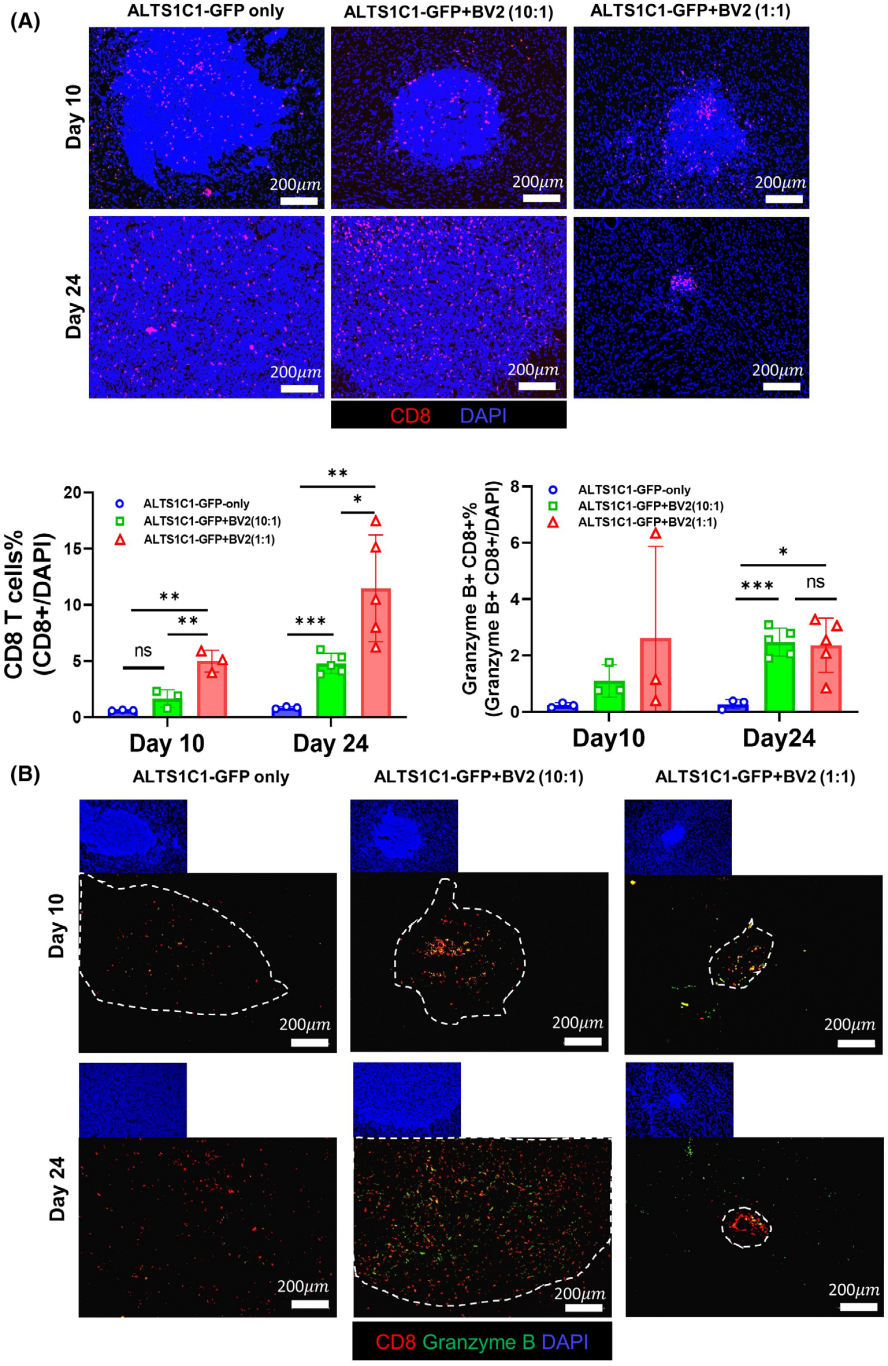
The presence of BV2 enhances T‐cell infiltration (A) Representative immunofluorescent images of ALTS1C1‐GFP astrocytoma in C57BL/6 mice from three groups—ALTS1C1‐GFP only, ALTS1C1‐GFP + BV2 (10 : 1), and ALTS1C1‐GFP + BV2 (1 : 1)—stained for CD8^+^ T cells (red) and nuclei (DAPI, blue). Scale bar = 200 μm. Brain tissues were collected on days 10 and 24 after tumor implantation. The proportion of infiltrating CD8^+^ T cells among DAPI^+^ cells within the tumor area is quantified and presented as mean ± SD (*n* ≥ 3 per group). Statistical analysis was performed using unpaired *t*‐tests (ns, not significant; *0.01 < *P* < 0.05, **0.001 < *P* < 0.01, and ***0.0001 < *P* < 0.001). CD8^+^ T cell infiltration was significantly increased in the BV2 co‐injection groups compared to the ALTS1C1‐GFP only group, particularly on day 24 post‐implantation. (B) Representative immunofluorescent images of ALTS1C1‐GFP astrocytoma in C57BL/6 mice from three groups—ALTS1C1‐GFP only, ALTS1C1‐GFP + BV2 (10 : 1), and ALTS1C1‐GFP + BV2 (1 : 1)—stained for CD8+ T cells (red), Granzyme B (green), and the nuclei (DAPI, blue). Tumor regions are outlined with white dotted lines. Scale bar = 200 μm. Brain tissues were collected on days 10 and 24 after tumor implantation. The proportion of CD8^+^ and Granzyme B^+^ cells among DAPI^+^ cells within the tumor area was quantified and presented as mean ± SD (*n* ≥ 3 per group). Statistical analysis was performed using unpaired *t*‐tests (ns, not significant; *0.01 < *P* < 0.05, and ***0.0001 < *P* < 0.001). BV2 co‐injection significantly increased the infiltration of CD8^+^ and Granzyme B^+^ cells compared to the ALTS1C1‐GFP only group.

The above results suggest a pivotal role of T cells in BV2‐mediated ALTS1C1 tumor growth delay. To further investigate if the presence of BV2 could affect T cell infiltration, the TME from day 10 and 24 tumors was examined by IF staining (Fig. 5A, Fig. S4A). The image of 10‐day tumors shows that ALTS1C1‐only tumors contain very few infiltrating CD8‐positive T cells (around 1% of DAPI‐positive cells) and sustain this low proportion when the tumor grows up to 24 days. On the other hand, the ratio of tumor‐infiltrating CD8+ T cells in BV2 co‐inoculated tumors, particularly the 1 : 1 ratio tumor, increased to around 4% on day 10 and further increased to around 11% on day 24. These results underscore the notion that BV2 serves as an attractant for CD8 T cells within the TME. To further elucidate the activation status of CD8 T cells, tissues were stained for the expression of functional marker Granzyme B (Fig. 5B). The IF staining data showed that just a few Granzyme B+ CD8+ T cells (around 0.2% of DAPI‐positive cells) infiltrated the 10‐day and 24‐day ALTS1C1‐only tumors. The number of Granzyme B+ CD8+ T cells rose to around 3% on day 10 in the group containing a 1 : 1 ratio of ALTS1Ca and BV2 cells, and the level was sustained to day 24. Notably, in the group inoculated with one‐tenth of BV2 cells, the Granzyme B+ CD8+ T cells also rose to 3% of DAPI‐positive cells.

### 3.5 The presence of BV2 sustains the level of circulating CD8 T cells and MDSC

**Fig. 6:**
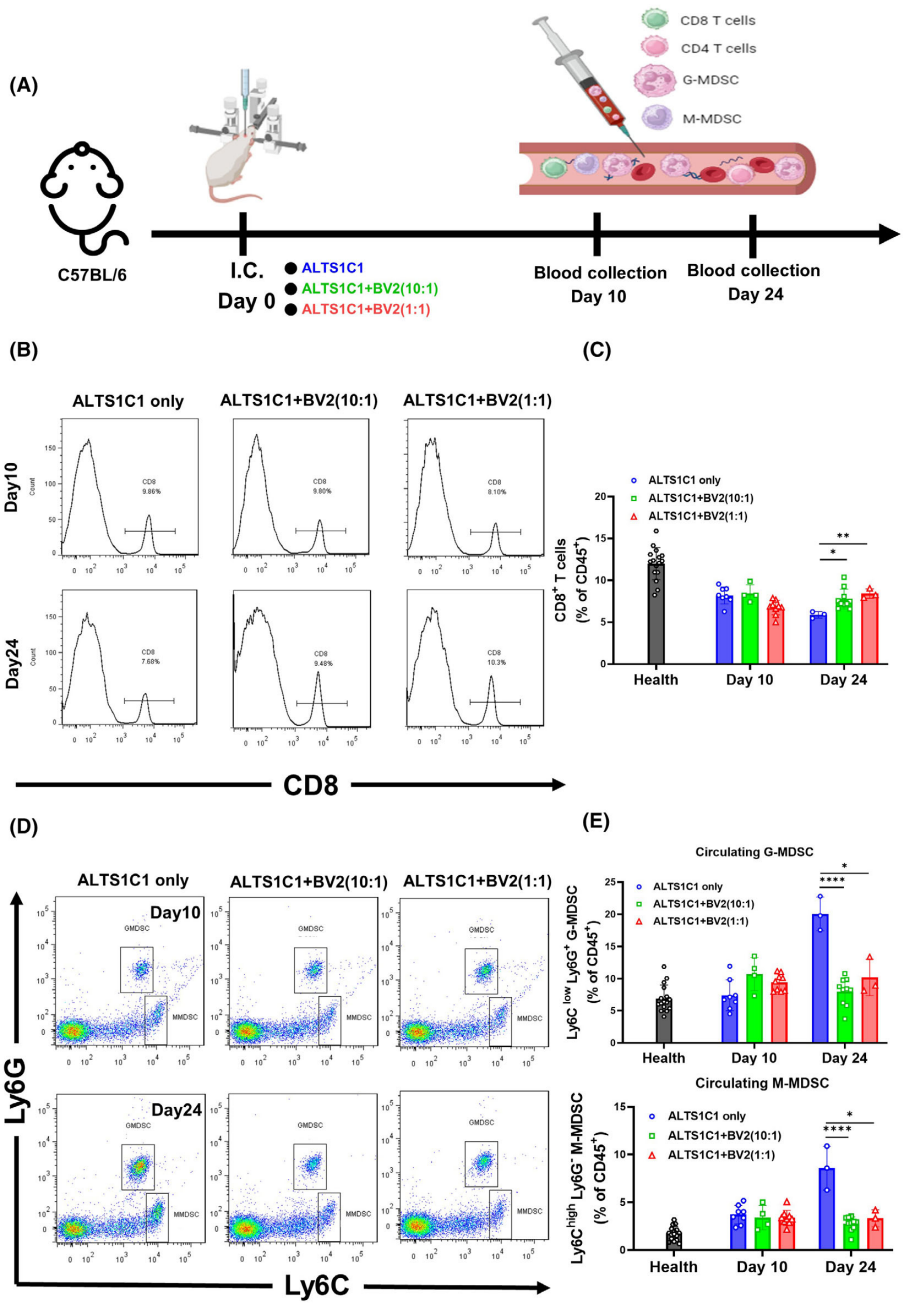
The presence of BV2 sustains the level of circulating CD8 T cells and MDSC (A) The timeline of ALTS1C1 and BV2 co‐implantation and the collection of circulating T cells and MDSCs. (B) Representative flow cytometry histograms showing circulating CD8^+^ T cells in C57BL/6 mice from three groups—ALTS1C1 only, ALTS1C1 + BV2 (10 : 1), and ALTS1C1 + BV2 (1 : 1)—at days 10 and 24 after tumor implantation. CD8^+^ T cells were identified by gating on CD8‐expressing lymphocytes. (C) Quantification of the proportion of circulating CD8^+^ T cells among CD45^+^ cells in C57BL/6 mice. Data are presented as mean ± SD (*n* ≥ 3 per group at each time point). Statistical analysis was performed using unpaired *t*‐tests (*0.01 < *P* < 0.05; **0.001 < *P* < 0.01). On day 24 after tumor implantation, BV2 co‐injections significantly increased the proportion of circulating CD8^+^ T cells within the CD45^+^ population compared to the ALTS1C1 only group. (D) Representative flow cytometry plots showing circulating MDSCs in C57BL/6 mice from three groups—ALTS1C1 only, ALTS1C1 + BV2 (10 : 1), and ALTS1C1 + BV2 (1 : 1)—at days 10 and 24 after tumor implantation. Granulocytic MDSCs (G‐MDSCs) were identified as Ly6C^low^ Ly6G^+^ cells, and monocytic MDSCs (M‐MDSCs) as Ly6C^high^ Ly6G^−^ cells. (E) Quantification of the proportion of circulating G‐MDSCs and M‐MDSCs among CD45^+^ cells in C57BL/6 mice. Data are presented as mean ± SD (*n* ≥ 3 per group at each time point). Statistical analysis was performed using unpaired *t*‐tests (*0.01 < *P* < 0.05, and ****P < 0.0001). On day 24 after tumor implantation, BV2 co‐injections significantly reduce the proportions of both circulating G‐MDSCs and M‐MDSCs within the CD45^+^ population compared to the ALTS1C1 only group.

The presence of BV2 enhances the Granzyme B+ CD8+ cells' infiltration into the brain TME. To explore whether this is correlated to changes in circulating immune cells, the blood was collected on the tenth and twenty‐fourth day after tumor implantation (Fig. 6A). The T cell populations were analyzed by flow cytometry using CD45 and CD8 antibodies (Fig. 6B). In healthy mice, circulating CD8 T cells constitute around 12% of CD45+ cells. In mice bearing ALTS1C1‐only tumors, this population decreases to 7.5% by day 10 and further to around 5% by day 24. Conversely, in mice with BV2‐containing ALTS1C1 tumors, the CD8+ T cell population remained at 7.5% (Fig. 6C). This finding suggests that BV2 presence alleviates immune suppression. We hypothesize that the immunosuppressive function of circulating MDSCs was mitigated in BV2‐containing ALTS1C1 tumor‐bearing mice. The sub‐populations of MDSCs in the circulating blood were also analyzed by flow cytometry using Ly6C and Ly6G antibodies. $\mathrm{Ly}6C^{\mathrm{low}}\mathrm{Ly}6G^{+}$ cells represent G‐MDSCs and $\mathrm{Ly}6C^{\text{high}}\mathrm{Ly}6G^{-}$ cells represent M‐MDSCs (Fig. 6D). G‐MDSCs comprise approximately 7.5% of CD45+ cells in healthy mice, rising slightly to around 9% in mice with BV2‐containing ALTS1C1 tumors. By day 24, G‐MDSCs surged to about 20% in mice bearing ALTS1C1‐only tumors, while remaining at about 9% in those with BV2‐containing tumors. A similar trend was observed with M‐MDSCs, which constituted approximately 4% of CD45+ cells in healthy mice. By day 10, M‐MDSCs increased by 2% compared to healthy mice and reached around 9% by day 24 in ALTS1C1‐only tumor‐bearing mice, but remained at 4% in BV2‐containing tumor‐bearing mice (Fig. 6E).

## 4 Discussion

This study used an *in vitro* coculture model to simulate early TME of tumor seeding or invasion, showing that tissue‐resident microglial cells‐BV2, rather than RAW264.7, can confine brain tumor cells into clusters, limiting their proliferation. This effect depends on the abundance of microglial cells, which act as guardians to restrict tumor spread and mediate the CD8 T cell recruitment with cytotoxic activity. However, tumor cells could escape this restriction when they become dominant. The study highlights the unique antitumor role of microglia at different phases of tumor progression.

Glioma is characterized by the extensive infiltration of myeloid cells, specifically microglia and macrophages [31]. These two types of cells are distributed throughout the tumor microenvironment (TME) in distinct spatial patterns. Microglia are primarily found at the edges of the tumor and in invasive islands, whereas infiltrating macrophages are predominantly located at the tumor core [32, 33, 34]. In our coculture system, BV2 microglia and IL‐4‐treated BMDMs occupied the cluster periphery and pushed tumor cells together, as shown in a time‐lapse video. This might explain why myeloid cells, particularly microglia and M2‐polarized macrophages, tend to migrate toward tumors and primarily localize at the tumor front. In contrast, peripheral macrophages like RAW264.7 and LPS‐treated BMDM do not form clusters with ALTS1C1 cells, suggesting their diverse role during tumor development. Moreover, BV2 specifically forms clusters with brain tumor cells, not with melanoma or pancreatic cells [29], highlighting their specific role in cluster formation in brain tumors. Previous studies have demonstrated that BV2 microglia can induce glioma cells to form tumor spheroids under normal cell culture conditions [29]. This study further compares microglial cells with other macrophage subsets, but the exact mechanism by which BV2 microglia and IL‐4‐treated BMDMs facilitate glioma cluster formation, in contrast to other macrophage subsets, remains unknown and warrants further investigation.

Reports show that glioma‐infiltrating microglia/macrophages promote glioma progression by stimulating proliferation and inhibiting apoptosis [35, 36]. In this coculture model, BV2 cells restrict ALTS1C1 proliferation mainly by reducing G1 and S phase cells and increasing G2/M phase cells. This restriction likely results from microglia gathering glioma cells, limiting space for growth. Unlike circulating tumor cell (CTC) clusters with high proliferation and metastatic potential [37] via plakoglobin‐mediated adhesion [38], microglia‐driven clustering physically constrains glioma growth.

Microglia play a crucial role in brain tumor immunity, exhibiting antitumor or pro‐tumor functions depending on their polarization states [12]. They are pivotal in tumor‐induced MHC I upregulation, facilitating antigen presentation and CD8+ T cell activation [39]. The animal experiments here demonstrate that BV2 co‐implanted tumors exhibit high CD8+ T cell infiltration and increased Granzyme B expression, indicating that tumor‐associated microglia can enhance T cell infiltration and cytotoxic activity, modulating antitumor immunity against gliomas. Glioma‐infiltrating microglia, particularly in the M2 state, can promote tumor progression through immunosuppression [12, 40]. BV2 cells express higher levels of M2‐associated genes and slightly elevated M1 markers compared to RAW264.7 cells. IL‐4‐treated BMDMs promote glioma clustering, unlike LPS‐treated BMDMs, suggesting that tumor‐associated BV2 cells were driven to an anti‐inflammatory phenotype that restricts proliferation while preserving antitumor immunity. These findings emphasize the complexity of microglia and the need for further research into their immunoregulatory mechanisms within the tumor microenvironment.

To assess the impact of microglial cells on tumor growth, we compared the growth of astrocytoma tumors by co‐implanting microglial cells with astrocytoma cells in the brains of both immune‐competent and immune‐compromised mice. The co‐implantation of microglial cells effectively restricted brain tumor growth in immunocompetent mice but not in immunocompromised mice. While tumor sizes were similar on day 10, a significant difference was observed by day 24. We noticed that the tumor size on day 10 was not significantly different in these two mice, and the most significant difference was on day 24. This finding indicates different roles of microglia at the initial versus the later stages of tumor development. At the beginning of tumor seeding, microglia play a role in restricting tumor cell proliferation. When tumor cells became dominant, microglia recruited T cells to restrain further or even kill tumor cells. The theory was partially supported by the finding that the co‐implantation of microglia increased the number of infiltrating CD8 T cells in immune‐competent mice and had more profound Granzyme B+ CD8 T cells at day 24. Moreover, when glioma developed in the presence of an equal number of microglial cells, it more rapidly induced higher expression of CD8^+^ Granzyme B^+^ T cells (approximately 3%), resulting in stronger tumor restriction. In contrast, the ALTS1C1‐GFP + BV2 (10 : 1) group reached the 3% level at a later time point, and the corresponding tumor suppression was weaker. The elevation of circulating CD8 T cells and reduction of MDSCs further certifies that BV2‐mediated antitumor immunity gradually becomes the major effect at the later tumor‐progressing stage.

For microglia to form tumor clusters and limit tumor progression effectively, their numbers must reach a critical ratio relative to tumor cells. *In vitro*, BV2 cells restrict ALTS1C1 growth, although the intensive gathering (cluster formation) was only observed under the ratio between 2 : 1 and 1 : 2. *In vivo*, a 1 : 1 ratio of glioma to microglial cells restricts tumor growth on days 10 and 24. However, when microglial numbers drop to one‐tenth of glioma cells, tumors evade restriction and grow unchecked. Without microglial replenishment, tumor growth remains unrestricted. These findings emphasize that the inherent microglial population is often insufficient to control tumor progression, stressing the need for adequate microglial abundance.

Our findings suggest that microglial cells possess intrinsic antitumor properties that could be harnessed for therapeutic purposes in glioma treatment. Translating this strategy into clinical application, however, presents several challenges, particularly regarding the sourcing and generation of therapeutic microglia. Since true microglia originate from yolk sac progenitors and are not readily accessible in adults, feasible alternatives include the *ex vivo* differentiation of autologous monocytes into microglia‐like cells or the generation of patient‐specific microglia from induced pluripotent stem cells (iPSCs). These approaches may reduce immunogenicity and ethical concerns associated with allogeneic transplantation. To further bridge the gap between experimental models and clinical therapy, future studies should incorporate glioma resection models in mice, followed by localized delivery of therapeutic microglia into the resection cavity to evaluate efficacy and safety. Ultimately, the development of scalable, human‐compatible microglia with stable antitumor phenotypes will be crucial for advancing microglia‐based therapies toward clinical application.

## 5 Conclusions

Microglia utilize two key strategies to limit brain tumor progression: suppressing tumor cell proliferation and recruiting T cells. The suppression of tumor cell proliferation occurs earlier than the recruitment of T cells, which takes more time to develop and may rely on assistance from circulating MDSCs. In summary, this study reveals that tumor‐associated microglia, rather than infiltrating macrophages, play a crucial role in restraining tumor growth. These findings also indicate that different strategies may be needed at various stages when designing treatment approaches for brain tumors.

## Conflict of interest

The authors declare no conflict of interest.

## Author contributions

T.‐C.S. designed, conducted the study, and drafted and revised the manuscript. C.‐F.Y. designed the study and revised the manuscript. S.‐Y.W. conducted the study. W.‐C.C. contributed to data analysis. C.‐S.C. designed the study and revised the manuscript. F.‐H.C. supervised the project and revised the manuscript.

## Peer review

The peer review history for this article is available at https://www.webofscience.com/api/gateway/wos/peer‐review/10.1002/1878‐0261.70102.

## Supporting information


**Fig. S1.** ALTS1C1 growth rate and cluster formation under co‐culture with BV2 or RAW264.7.
**Fig. S2.** Phenotype analysis of macrophage cells.
**Fig. S3.** *In vivo* tumor growth of ALTS1C1 and ALTS1C1‐GFP cells.
**Fig. S4.** CD4 infiltrates in tumor microenvironment.

## Data Availability

The data that supports the findings of this study are available in figures and the Supporting Information of this article.

## References

[1] Price M , Ballard C , Benedetti J , Neff C , Cioffi G , Waite KA , et al. CBTRUS statistical report: primary brain and other central nervous system tumors diagnosed in the United States in 2017–2021. Neuro‐Oncology. 2024;26(Supplement_6):vi1–vi85. 39371035 10.1093/neuonc/noae145 PMC11456825

[2] Louis DN , Perry A , Wesseling P , Brat DJ , Cree IA , Figarella‐Branger D , et al. The 2021 WHO classification of tumors of the central nervous system: a summary. Neuro‐Oncology. 2021;23(8):1231–1251. 34185076 10.1093/neuonc/noab106 PMC8328013

[3] Noiphithak R , Veerasarn K . Clinical predictors for survival and treatment outcome of high‐grade glioma in Prasat neurological institute. Asian J Neurosurg. 2017;12(1):28–33. 28413528 10.4103/1793-5482.148791 PMC5379799

[4] Mehraj U , Dar AH , Wani NA , Mir MA . Tumor microenvironment promotes breast cancer chemoresistance. Cancer Chemother Pharmacol. 2021;87(2):147–158. 33420940 10.1007/s00280-020-04222-w

[5] Zheng X , Weigert A , Reu S , Guenther S , Mansouri S , Bassaly B , et al. Spatial density and distribution of tumor‐associated macrophages predict survival in non‐small cell lung carcinoma. Cancer Res. 2020;80(20):4414–4425. 32699134 10.1158/0008-5472.CAN-20-0069

[6] Cassetta L , Pollard JW . A timeline of tumour‐associated macrophage biology. Nat Rev Cancer. 2023;23(4):238–257. 36792751 10.1038/s41568-022-00547-1

[7] Colonna M , Butovsky O . Microglia function in the central nervous system during health and neurodegeneration. Annu Rev Immunol. 2017;35:441–468. 28226226 10.1146/annurev-immunol-051116-052358 PMC8167938

[8] Lee E , Eo JC , Lee C , Yu JW . Distinct features of brain‐resident macrophages: microglia and non‐parenchymal brain macrophages. Mol Cells. 2021;44(5):281–291. 33972475 10.14348/molcells.2021.0060 PMC8175151

[9] Mass E , Nimmerjahn F , Kierdorf K , Schlitzer A . Tissue‐specific macrophages: how they develop and choreograph tissue biology. Nat Rev Immunol. 2023;23(9):563–579. 36922638 10.1038/s41577-023-00848-y PMC10017071

[10] Lawson LJ , Perry VH , Dri P , Gordon S . Heterogeneity in the distribution and morphology of microglia in the normal adult mouse brain. Neuroscience. 1990;39(1):151–170. 2089275 10.1016/0306-4522(90)90229-w

[11] Borst K , Dumas AA , Prinz M . Microglia: Immune and non‐immune functions. Immunity. 2021;54(10):2194–2208. 34644556 10.1016/j.immuni.2021.09.014

[12] Wu SY , Watabe K . The roles of microglia/macrophages in tumor progression of brain cancer and metastatic disease. Front Biosci. 2017;22(10):1805–1829. 10.2741/4573 PMC5658785 28410147

[13] Cole AP , Hoffmeyer E , Chetty SL , Cruz‐Cruz J , Hamrick F , Youssef O , et al. Microglia in the brain tumor microenvironment. Adv Exp Med Biol. 2020;1273:197–208. 33119883 10.1007/978-3-030-49270-0_11

[14] da Fonseca AC , Amaral R , Garcia C , Geraldo LH , Matias D , Lima FR . Microglia in cancer: for good or for bad? Adv Exp Med Biol. 2016;949:245–261. 27714693 10.1007/978-3-319-40764-7_12

[15] Kees T , Lohr J , Noack J , Mora R , Gdynia G , Tödt G , et al. Microglia isolated from patients with glioma gain antitumor activities on poly (I:C) stimulation. Neuro‐Oncology. 2012;14(1):64–78. 22015597 10.1093/neuonc/nor182 PMC3245995

[16] Mills CD , Kincaid K , Alt JM , Heilman MJ , Hill AM . M‐1/M‐2 macrophages and the Th1/Th2 paradigm. J Immunol. 2000;164(12):6166–6173. 10843666 10.4049/jimmunol.164.12.6166

[17] Yao Y , Xu XH , Jin L . Macrophage polarization in physiological and pathological pregnancy. Front Immunol. 2019;10:792. 31037072 10.3389/fimmu.2019.00792 PMC6476302

[18] Mantovani A , Sozzani S , Locati M , Allavena P , Sica A . Macrophage polarization: tumor‐associated macrophages as a paradigm for polarized M2 mononuclear phagocytes. Trends Immunol. 2002;23(11):549–555. 12401408 10.1016/s1471-4906(02)02302-5

[19] Li M , Jiang P , Wei S , Wang J , Li C . The role of macrophages‐mediated communications among cell compositions of tumor microenvironment in cancer progression. Front Immunol. 2023;14:1113312. 36845095 10.3389/fimmu.2023.1113312 PMC9947507

[20] Cassetta L , Pollard JW . Targeting macrophages: therapeutic approaches in cancer. Nat Rev Drug Discov. 2018;17(12):887–904. 30361552 10.1038/nrd.2018.169

[21] van Dalen FJ , van Stevendaal MHME , Fennemann FL , Verdoes M , Ilina O . Molecular repolarisation of tumour‐associated macrophages. Molecules. 2018;24:1. 30577495 10.3390/molecules24010009 PMC6337345

[22] Michel M . The M1/M2 immune polarization concept in microglia: a fair transfer? Neuroimmunol Neuroinflammation. 2014;1:6–7.

[23] Durafourt BA , Moore CS , Zammit DA , Johnson TA , Zaguia F , Guiot MC , et al. Comparison of polarization properties of human adult microglia and blood‐derived macrophages. Glia. 2012;60(5):717–727. 22290798 10.1002/glia.22298

[24] Tu S , Lin X , Qiu J , Zhou J , Wang H , Hu S , et al. Crosstalk between tumor‐associated microglia/macrophages and CD8‐positive T cells plays a key role in glioblastoma. Front Immunol. 2021;12:650105. 34394072 10.3389/fimmu.2021.650105 PMC8358794

[25] Wang SC , Hong JH , Hsueh C , Chiang CS . Tumor‐secreted SDF‐1 promotes glioma invasiveness and TAM tropism toward hypoxia in a murine astrocytoma model. Lab Investig. 2012;92(1):151–162. 21894147 10.1038/labinvest.2011.128

[26] Henn A , Lund S , Hedtjärn M , Schrattenholz A , Pörzgen P , Leist M . The suitability of BV2 cells as alternative model system for primary microglia cultures or for animal experiments examining brain inflammation. ALTEX. 2009;26(2):83–94. 19565166 10.14573/altex.2009.2.83

[27] Raschke WC , Baird S , Ralph P , Nakoinz I . Functional macrophage cell lines transformed by Abelson leukemia virus. Cell. 1978;15(1):261–267. 212198 10.1016/0092-8674(78)90101-0

[28] Hartley JW , Evans LH , Green KY , Naghashfar Z , Macias AR , Zerfas PM , et al. Expression of infectious murine leukemia viruses by RAW264.7 cells, a potential complication for studies with a widely used mouse macrophage cell line. Retrovirology. 2008;5:1. 18177500 10.1186/1742-4690-5-1 PMC2253558

[29] Wu SY , Yu WJ , Chien TY , Ren YA , Chen CS , Chiang CS . Microglia‐mediated drug substance transfer promotes chemoresistance in brain tumors: insights from an *in vitro* co‐culture model using GCV/Tk prodrug system. Cancer Cell Int. 2024;24(1):35. 38238749 10.1186/s12935-024-03213-8 PMC10795391

[30] Martinez FO , Gordon S , Locati M , Mantovani A . Transcriptional profiling of the human monocyte‐to‐macrophage differentiation and polarization: new molecules and patterns of gene expression. J Immunol. 2006;177(10):7303–7311. 17082649 10.4049/jimmunol.177.10.7303

[31] Coniglio SJ , Segall JE . Review: molecular mechanism of microglia stimulated glioblastoma invasion. Matrix Biol. 2013;32(7–8):372–380. 23933178 10.1016/j.matbio.2013.07.008

[32] Wang SC , Yu CF , Hong JH , Tsai CS , Chiang CS . Radiation therapy‐induced tumor invasiveness is associated with SDF‐1‐regulated macrophage mobilization and vasculogenesis. PLoS One. 2013;8(8):e69182. 23940516 10.1371/journal.pone.0069182 PMC3734136

[33] Evans KT , Blake K , Longworth A , Coburn MA , Insua‐Rodríguez J , McMullen TP , et al. Microglia promote anti‐tumour immunity and suppress breast cancer brain metastasis. Nat Cell Biol. 2023;25(12):1848–1859. 37957324 10.1038/s41556-023-01273-y PMC11414741

[34] Kettenmann H , Hanisch UK , Noda M , Verkhratsky A . Physiology of microglia. Physiol Rev. 2011;91(2):461–553. 21527731 10.1152/physrev.00011.2010

[35] Zhai H , Heppner FL , Tsirka SE . Microglia/macrophages promote glioma progression. Glia. 2011;59(3):472–485. 21264953 10.1002/glia.21117 PMC3080032

[36] Arcuri C , Fioretti B , Bianchi R , Mecca C , Tubaro C , Beccari T , et al. Microglia‐glioma cross‐talk: a two way approach to new strategies against glioma. Front Biosci. 2017;22(2):268–309. 10.2741/4486 27814616

[37] Aceto N . Bring along your friends: homotypic and heterotypic circulating tumor cell clustering to accelerate metastasis. Biom J. 2020;43(1):18–23. 10.1016/j.bj.2019.11.002 PMC7090281 32200952

[38] Aceto N , Bardia A , Miyamoto DT , Donaldson MC , Wittner BS , Spencer JA , et al. Circulating tumor cell clusters are oligoclonal precursors of breast cancer metastasis. Cell. 2014;158(5):1110–1122. 25171411 10.1016/j.cell.2014.07.013 PMC4149753

[39] Chang CY , Jeon SB , Yoon HJ , Choi BK , Kim SS , Oshima M , et al. Glial TLR2‐driven innate immune responses and CD8(+) T cell activation against brain tumor. Glia. 2019;67(6):1179–1195. 30720218 10.1002/glia.23597

[40] Kaur G , Han SJ , Yang I , Crane C . Microglia and central nervous system immunity. Neurosurg Clin N Am. 2010;21(1):43–51. 19944965 10.1016/j.nec.2009.08.009

